# Preliminary Study of Subclinical Brain Alterations in Patients With Asymptomatic Carotid Vulnerable Plaques Using Intravoxel Incoherent Motion Imaging by Voxelwise Comparison: A Study of Whole-Brain Imaging Measures

**DOI:** 10.3389/fnins.2020.562830

**Published:** 2020-12-15

**Authors:** Jiuqing Guo, Lirong OuYang, Xiaoyi Wang, Weihua Liao, Qing Huang, Wei He, Gaofeng Zhou, Shuai Yang

**Affiliations:** ^1^Department of Radiology, Xiangya Hospital, Central South University, Changsha, China; ^2^Department of Neurology, Xiangya Hospital, Central South University, Changsha, China

**Keywords:** multi-*b*-value DWI, double-exponential model, carotid artery, vulnerable plaque, intravoxel incoherent motion imaging

## Abstract

**Objective:**

To preliminarily explore subclinical brain alterations in an asymptomatic carotid vulnerable plaque group based on intravoxel incoherent motion (IVIM) imaging through voxelwise comparison in the whole brain.

**Materials and Methods:**

Forty-nine elderly participants underwent multi-*b*-value DWI, of whom 24 participants with asymptomatic carotid vulnerable plaques and <50% stenosis served as the test group, while the rest served as the healthy control group. After fitting the double-exponential model, slow ADC (Ds) and the fraction of fast ADC (*f*) values of the whole brain were obtained, which then were compared in a voxelwise manner by two-sample *t*-test. Multiple comparisons were corrected by the family-wise error (FWE) method with a corrected threshold of *P* < 0.05. Pearson correlations between IVIM parameters in altered brain regions and blood pressure, glucose, lipid, and homocysteine were calculated.

**Results:**

For the test group, the *Z*-normalized Ds values were significantly higher in the left median cingulate and paracingulate gyrus (DCG.L), posterior cingulate gyrus (PCG. L), and left precuneus gyrus (PCUN.L) (cluster size = 156) and in the left middle frontal gyrus (MFG.L), orbital middle frontal gyrus (ORBmid.L), and superior frontal gyrus (SFG.L) (cluster size = 165); the *Z*-normalized Ds values were significantly lower in the right middle temporal gyrus (MTG.R) and inferior temporal gyrus (ITG.R) (cluster size = 116); and the *Z*-normalized *f*-values were significantly lower in the MTG.R and ITG.R (cluster size = 85) (*p* < 0.05, FWE correction). LDL-C was negatively correlated with the *Z*-normalized Ds values in the DCG.L, PCG.L, and PCUN.L (*r* = 0.601, *p* = 0.002). LDL-C was positively correlated with the *Z*-normalized *f*-value in the MTG.R and ITG.R (*r* = 0.405, *p* = 0.05). Systolic blood pressure was positively correlated with the *Z*-normalized Ds values in the MFG.L, ORBmid.L, and SFG.L (*r* = 0.433, *p* = 0.035).

**Conclusion:**

This study was the first to detect subclinical brain alterations in asymptomatic carotid vulnerable plaque group through IVIM using whole-brain voxelwise comparisons, which were partially correlated with blood pressure and lipids. Thus, IVIM might be utilized as a noninvasive biomarker of microvascular and microstructural brain changes in the asymptomatic carotid vulnerable plaque group.

## Introduction

Asymptomatic carotid artery stenosis is common in the general population; its prevalence is 6% in elderly male people and 4.4% in elderly female people (de Weerd et al., 2010). Patients with asymptomatic carotid artery stenosis without recent neurological symptoms have an increased risk of ischemic stroke, especially in the ipsilateral carotid artery region (Halliday et al., 2010). Carotid endarterectomy has a much lower efficiency in reducing the risk of stroke in asymptomatic patients than in symptomatic patients (Halliday et al., 2004). This difference in absolute risk reduction after carotid endarterectomy suggests the importance of plaque vulnerability, except for plaque size and lumen occlusion. Several clinical studies showed that in addition to the degree of carotid artery stenosis, the thickness of the carotid intima-media and the size of the hypoechoic area in plaques reflect the vulnerability of carotid plaques, which is closely associated with the occurrence of cerebral infarction (Moroni et al., 2016). Carotid plaques are associated with white matter lesions and resting infarction. Although no causal correlation has been identified from his association, the existence of carotid plaques may be regarded as an important risk factor for subclinical brain injury (Moroni et al., 2016).

Although some studies have shown that carotid plaques are related to changes in cerebral diffusion or perfusion (Gao et al., 2009; Rowe Bijanki et al., 2013; Moroni et al., 2016; Liu et al., 2020), few studies have combined diffusion and perfusion in the same patient. Diffusion-weighted imaging (DWI) is a functional magnetic resonance imaging (MRI) technique that can assess the microscopic motion of water protons in tissues. The apparent diffusion coefficient (ADC) can quantitatively reflect the diffusivity. The monoexponential model is widely used in DWI. Some researchers have found that with an increase in the *b*-value, the signal of DWI is no longer linear but shows double exponential characteristics (Kwee et al., 2010). To obtain information on perfusion, another functional MRI technique, called intravoxel incoherent motion (IVIM), can supply information about both tissue diffusion and perfusion components. IVIM uses multiple *b*-values and a biexponential signal model to enable quantitative parameters that can separately reflect tissue microcapillary perfusion and tissue microstructure and could potentially provide a noninvasive tool for monitoring tissue microvascular growth and degeneration (Finkenstaedt et al., 2017). The IVIM model ascribes the signal attenuation in diffusion-weighted images to two main components: a slow component that is due to water self-diffusion, i.e., the transport of water molecules within tissue as a result of random molecular movements, and a fast component that is due to the flow of water molecules in segments of the capillary network (Le Bihan et al., 1986, 1988). In the brain, the IVIM model has been used to quantify changes in perfusion or diffusion in the presence of disease, such as tumors and acute stroke (Iima and Bihan, 2016; Keil et al., 2017). However, there is a lack of study on diffusion functional MRI based on IVIM compared to classic functional MRI, such as resting-state fMRI, diffusion tensor imaging (DTI), and artery spin labeling (ASL).

Carotid vulnerable plaques may be considered a marker of higher ischemic or cognitive vulnerability of the brain, which may allow clinicians to identify subjects at risk of stroke and cognitive impairments and prompt an aggressive correction of cardiovascular risk factors, eventually including the initiation of treatment. This study aimed to investigate subclinical brain alterations in patients with asymptomatic carotid vulnerable plaques using biexponential model parameters [slow ADC (Ds) and fraction of fast ADC (f)] through voxelwise comparison in the whole brain and to determine whether IVIM reflects subclinical brain alterations at an early stage in patients with asymptomatic carotid vulnerable plaques.

## Materials and Methods

This prospective observational study was approved by the ethical standards committee on human experimentation at Central South University (CSU), and all patients signed written informed consent. This study was a part of the National Health Commission’s stroke screening and prevention program.

### Study Participants

**FIGURE 1:**
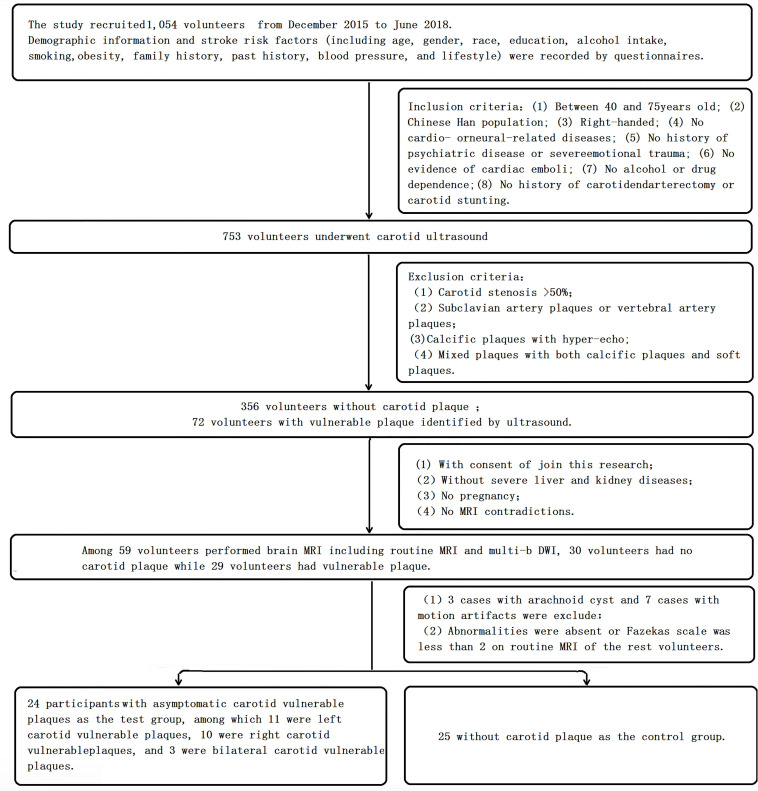
Flowchart.

The study recruited 1,054 volunteers from the Wujialing community in Changsha city, Hunan Province, China, from December 2015 to June 2018. Demographic information and stroke risk factors (including age, sex, race, education, alcohol intake, smoking, obesity, family history, past history, blood pressure, and lifestyle) were recorded using questionnaires. The inclusion criteria were as follows: (1) between 40 and 75 years old; (2) Chinese Han population; (3) right-handed; (4) no cardiac or neurological diseases; (5) no history of psychiatric disease or severe emotional trauma; (6) no evidence of cardiac emboli; (7) no alcohol or drug dependence; and (8) no history of carotid endarterectomy or carotid stunting. Volunteers with atrial fibrillation, cardiovascular disease, neurologic disease, psychosis, psychological trauma, cardiogenic embolism, or alcohol or drug dependence were excluded. Then,753 volunteers underwent carotid ultrasound. Volunteers with >50% carotid stenosis, subclavian artery plaques or vertebral artery plaques, calcific plaques with hyperecho, and mixed plaques with both calcific plaques and soft plaques were excluded. Volunteers without contraindications for MRI, without severe liver and kidney diseases, who were not pregnant, and who provided consent to join the study underwent brain MRI, including routine MRI and multi-*b*-value DWI. Three patients with arachnoid cysts and seven patients with motion artifacts were excluded. Abnormalities were absent or the Fazekas scale was less than two based on routine MRIs of the remaining volunteers. After screening based on the inclusion and exclusion criteria, a total of 49 participants were ultimately recruited and entered the final analysis. The flowchart is shown in Figure 1.

### Blood Collection

Fasting venous blood was collected from an elbow vein. Blood glucose, total cholesterol, triglycerides, high-density lipoprotein cholesterol (HDL-C), low-density lipoprotein cholesterol (LDL-C), and homocysteine (Hcy) were measured by the Department of Laboratory Medicine, Xiangya Hospital, Center South University. Blood pressure (systolic and diastolic blood pressure) was recorded.

### Carotid Ultrasound Acquisition and Analysis

The presence of plaques in the same region of the left and right sides of the common carotid artery [common carotid arteries (CCAs)] and bifurcation was detected by ultrasound. Carotid ultrasound was performed by a sonographer using a Philips 5-MHz Sector Array transducer (IU22, Philips Ultrasound, Bothell, WA, United States). All measurements were obtained using the grayscale median (GSM) (Elatrozy et al., 1998). The plaques were divided into three types: hypoechoic, isoechoic, or hyperechoic. The plaques were hypoechoic or isoechoic, the plaque echo was mixed, and the plaque surface was irregular or ulcerated, which is the standard for the definition of vulnerable plaques identified by color Doppler ultrasound of the carotid artery (Gray-Weale et al., 1988; Elatrozy et al., 1998). Among the 49 participants, 24 with asymptomatic carotid vulnerable plaques but <50% carotid artery stenosis were included in the test group; among these patients, 11 had left carotid vulnerable plaques, 10 had right carotid vulnerable plaques, and three had bilateral carotid vulnerable plaques. Twenty-five participants without carotid vulnerable plaques were used as the control group (see flowchart, Figure 1).

### IVIM-MRI Imaging Acquisition

Images were acquired on a 3-T MRI scanner (Signa HDx; General Electric Healthcare, Milwaukee, WI, United States) with an eight-channel head coil. The MRI sequences included 3D T1-weighted imaging, T2-weighted imaging, T2-weighted fluid attenuated inversion recovery imaging (T2/FLAIR), and multi-*b*-value DWI. Structural images were acquired using an axial 3D brain volume imaging (3D-BRAVO) sequence. The parameters were as follows: voxel size = 1 × 1 × 1 mm^3^; section thickness = 1 mm; repetition time (TR) = 8 ms, echo time (TE) = 3 ms; inversion time (TI) = 800 ms; flip angle = 7°; and field of view (FOV) = 256 × 256 mm^2^. The IVIM DWI was performed with a single-shot diffusion-weighted spin-echo echo-planar sequence using 20 different *b*-values: *b* = 0, 10, 30, 50, 80, 100, 120, 150, 200, 300, 500, 800, 1000, 1300, 1500, 1800, 2400, 3000, 3600, and 4500 s/mm^2^. The following MR imaging parameters were used: TR = 5600 ms; TE = 105 ms; imaging matrices = 256 × 256; slice thickness = 4 mm; axial slices = 28; gap = 5 mm; and two excitations.

### Image Analysis

**FIGURE 2:**
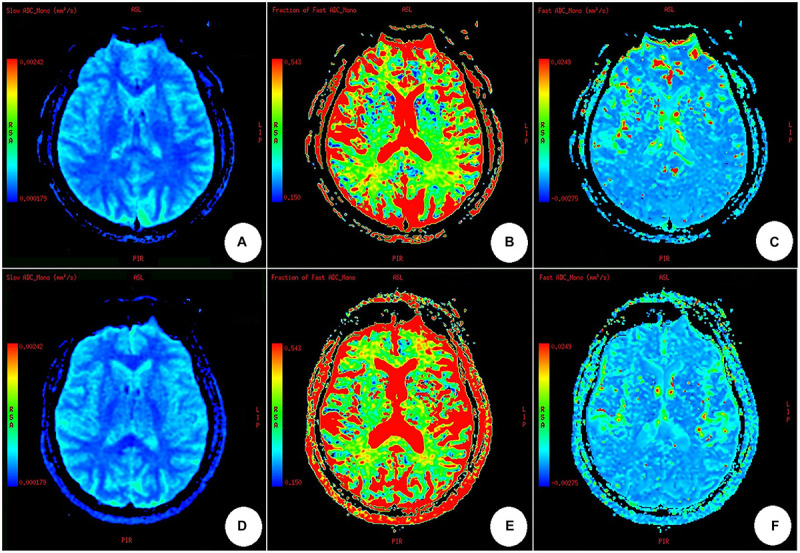
Maps of slow ADC (Ds), fraction of fast ADC (f), and fast ADC (Df) through IVIM fitting. **(A–C)** A healthy participant; **(D–F)** a participant with carotid vulnerable plaques. There was no difference among the IVIM parameters obtained from a healthy subject **(A–C)** and a participant with carotid artery plaques **(D–F)**, nor was there any difference between the bilateral cerebral hemispheres in **(A–F)**, by naked eye observation.

All data for processing were transferred to a workstation (Advantage Windows Workstation 4.4; GE Healthcare) to produce the IVIM parameters (Ds, f with a two-segment mono-exponential fitting with a *b*-value threshold of 200 s/mm^2^ separating the diffusion and perfusion effects, using all *b*-values in the following equation: *s*(*b*)/*s*0 = *fe*^–^^b^^*Df^ + (1 - *f*)*e*^–^^b^^*Ds^). Maps of the Ds, *f*, and Df values were obtained (Figure 2); however, based on the study of Wen-Chau Wu et al. (2015), who stated that D^∗^-Df in this study-has limited robustness and should be interpreted with caution, Df analysis was not conducted in this study. Five circular regions of interest (ROIs) in five locations of the ipsilateral cerebral hemisphere of carotid vulnerable plaques-ACA territory, MCA territory, PCA territory, anterior limb of internal capsule, and posterior limb of internal capsule-were delineated by an experienced radiologist (with 5 years of experience in neuroimaging), and the same was performed in the opposite hemisphere to obtain the Ds values and *f*-values. The ROIs were values less than the analyzed anatomical structure size and were limited to 20–40 pixels, avoiding blood vessels and cerebrospinal fluid. The ROIs were delineated again with approximately the same size after 30 days by the same radiologist.

**FIGURE 3:**
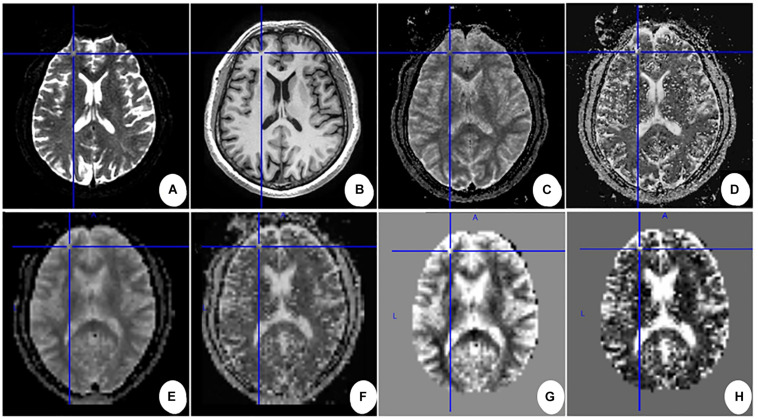
Main data pre-processing work flow for one subject. **(A)** First b0-volume; **(B–D)** 3D T1 images, Ds maps, and *f* maps after being coregistered to the first b0-volume. **(E,F)** Ds maps and *f* maps in the MNI space after normalizing. **(G,H)** The *Z*-normalized Ds maps and *f* maps after removing the skull. Cross lines are shown to help comparison between images.

Data preprocessing was performed using SPM12 (Statistical Parametric Mapping, Wellcome Trust Center for Neuroimaging,^1^) in MATLAB 2013b (MathWorks, Natick, MA, United States). First, we coregistered the 3D T1 images and the parametric maps (Ds maps and *f* maps) to the first b0-volume. Second, for each subject, the transformation matrix was calculated by nonlinear registration of the coregistered 3D T1 images to the tissue probability maps in the Montreal Neurological Institute (MNI) space and then applied to normalize the parametric maps into the MNI space. Third, the normalized parametric maps were standardized using the *z*-standardization method (De Luca et al., 2019). The method was as follows: (1) calculate the global mean and standard deviation across voxels for the parametric map; (2) subtract the global mean from the value in each voxel and divide the value in each voxel by the standard deviation. Finally, the standardized parametric maps were spatially smoothed with a Gaussian kernel of 8 mm × 8 mm × 8 mm FWHM. The main data preprocessing workflow is shown in Figure 3.

### Statistical Analysis

Statistical analysis was performed using the SPSS 23.0 statistical package (SPSS Version 23.0; IBM, Armonk, NY, United States). The measurement data are presented as the mean ± standard deviation (SD), and the enumeration data are presented as a proportion (%). The independent sample *t*-test or Wilcoxon rank sum test was used for comparison of measurement data between groups, and the chi-square test or Fisher’s test was used for comparison of enumeration data between groups. The ROI was measured twice at different times by the same radiologist. Interobserver agreement was assessed using the interclass correlation coefficient (ICC). A paired, two-tailed *t*-test was used to assess the Ds values and *f*-values of the cerebral region between the ipsilateral hemisphere of unilateral carotid vulnerable plaques and the contralateral hemisphere. Two-sample *t*-tests were used to assess whole-brain parameters using SPM12 software (cluster level *P* < 0.05, FWE correction) based on MATLAB 2013b, with age and sex as covariates. Pearson correlation was used to evaluate the relationship between the Ds values and *f*-values of the altered cerebral regions and systolic blood pressure, diastolic blood pressure, blood glucose, total cholesterol, triglycerides, HDL-C, LDL-c, and Hcy. *P* < 0.05 was considered to indicate statistical significance.

## Results

A total of 49 participants underwent MRI scans, 24 participants with asymptomatic carotid vulnerable plaques and 25 participants without carotid vulnerable plaques. The baseline characteristics of the patients with asymptomatic carotid vulnerable plaques and the controls are summarized in Table 1. The asymptomatic carotid vulnerable plaque and control groups did not significantly differ with respect to age, sex, hypertension, hyperlipidemia, diabetes mellitus, smoking, lack of exercise, body mass index (BMI), family history of stroke, systolic blood pressure, diastolic blood pressure, blood glucose, total cholesterol, triglycerides, HDL-C, LDL-C, or Hcy.

**TABLE 1:** Baseline characteristics of patients with asymptomatic carotid vulnerable plaques and controls.

	Asymptomatic carotid vulnerable plaque group (*n* = 24)	Control group (*n* = 25)	*p*-value
Mean age (years)	627	597	0.263
Sex (male), *n* (%)	11 (45.8%)	11 (44.0%)	0.897
Hypertension, *n* (%)	12 (50.0%)	11 (44.0%)	0.674
Hyperlipemia, *n* (%)	8 (33.3%)	7 (28.0%)	0.686
Diabetes, *n* (%)	1 (4.2%)	3 (12.0%)	0.317
Smoking, *n* (%)	7 (29.2%)	8 (32.0%)	0.83
Lack of exercises, *n* (%)	7 (29.2%)	8 (32.0%)	0.83
BMI	26.233.92	27.413.52	0.57
Family history of stroke, *n* (%)	14 (58.3%)	15 (60.0%)	0.906
Systolic blood pressure (mmHg)	61.757.37	59.486.65	0.263
Diastolic blood pressure (mmHg)	140.4218.88	135.3214.22	0.29
Blood glucose (HbA1c)	86.7510.60	84.3611.17	0.446
Triglycerides	5.671.06	5.821.04	0.616
Total cholesterol	2.141.18	2.020.85	0.678
HDL-C	5.171.19	5.760.78	0.053
LDL-C	1.380.52	1.410.44	0.833
Hcy	3.540.98	3.580.68	0.876

*Data are presented as the mean ± standard deviation. Body mass index (BMI), high-density lipoprotein cholesterol (HDL-C), low density lipoprotein cholesterol (LDL-c), homocysteine (Hcy).*

For 11 participants with left carotid vulnerable plaques and 10 participants with right carotid vulnerable plaques, the reproducibility of the size of ROIs and the first and second measurement of similar locations of ACA territory, MCA territory, PCA territory, anterior limb of internal capsule, and posterior limb of internal capsule were excellent, with ICC >0.8.

The Ds value and *f*-values of five locations of the brain (ACA territory, MCA territory, PCA territory, anterior limb of internal capsule, and posterior limb of internal capsule) between the ipsilateral hemisphere of unilateral carotid vulnerable plaques and the contralateral hemisphere were not significantly different (*P* > 0.05) (Table 2).

**TABLE 2:** Comparison between the corresponding side of the hemisphere and the contralateral hemisphere of 21 patients with unilateral carotid vulnerable plaques.

	Ipsilateral cerebral hemisphere of carotid vulnerable plaques	Contralateral cerebral hemisphere of carotid vulnerable plaques	*p*-value
**Ds (mm^2^/s)**			
ACA territory	0.0004650.000063	0.0004600.000055	0.808
MCA territory	0.0005110.000047	0.0005210.000065	0.490
PCA territory	0.0004410.000053	0.0004420.000052	0.94
Anterior limb of internal capsule	0.0004610.000035	0.0004510.000031	0.289
Posterior limb of internal capsule	0.0003380.000043	0.0003430.000044	0.627
***f***			
ACA territory	0.4320.074	0.4420.080	0.634
MCA territory	0.4490.055	0.4430.079	0.809
PCA territory	0.3720.070	0.3710.058	0.947
Anterior limb of internal capsule	0.4170.063	0.4410.119	0.479
Posterior limb of internal capsule	0.4280.103	0.4230.075	0.821

*Data are presented as the mean ± standard deviation. Anterior cerebral artery (ACA), middle cerebral artery (MCA), and posterior cerebral artery (PCA).*

**FIGURE 4:**
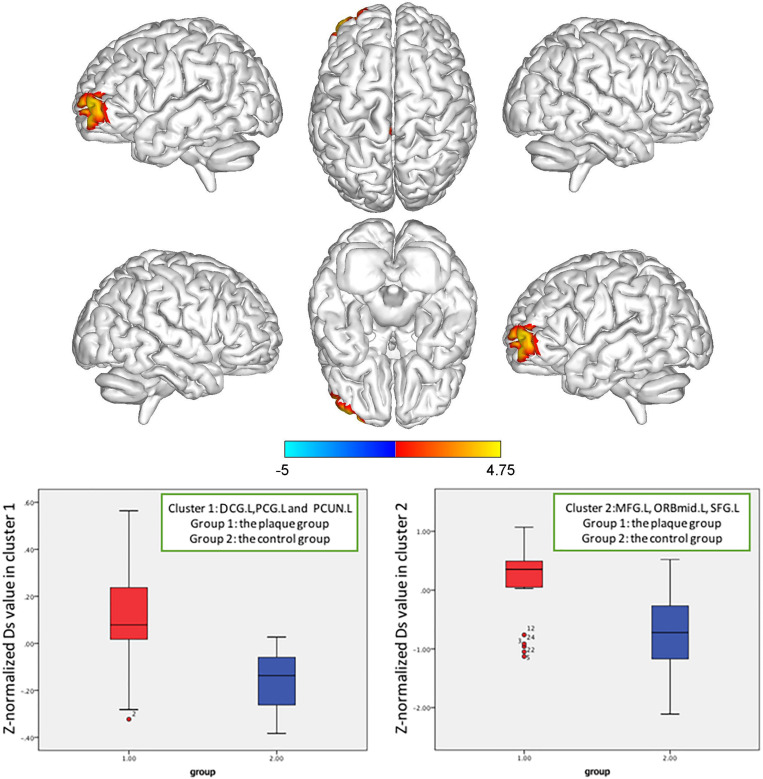
*Z*-normalized Ds value alterations in the asymptomatic carotid vulnerable plaque group. Significantly increased Ds values in the left median cingulate and paracingulate gyrus (DCG.L), posterior cingulate gyrus (PCG.L) and left precuneus gyrus (PCUN.L) (cluster size = 156), left middle frontal gyrus (MFG.L), orbital middle frontal gyrus (ORBmid.L), and superior frontal gyrus (SFG.L) (cluster size = 165) (*p* < 0.05, FWE correction) compared to the control group.

**FIGURE 5:**
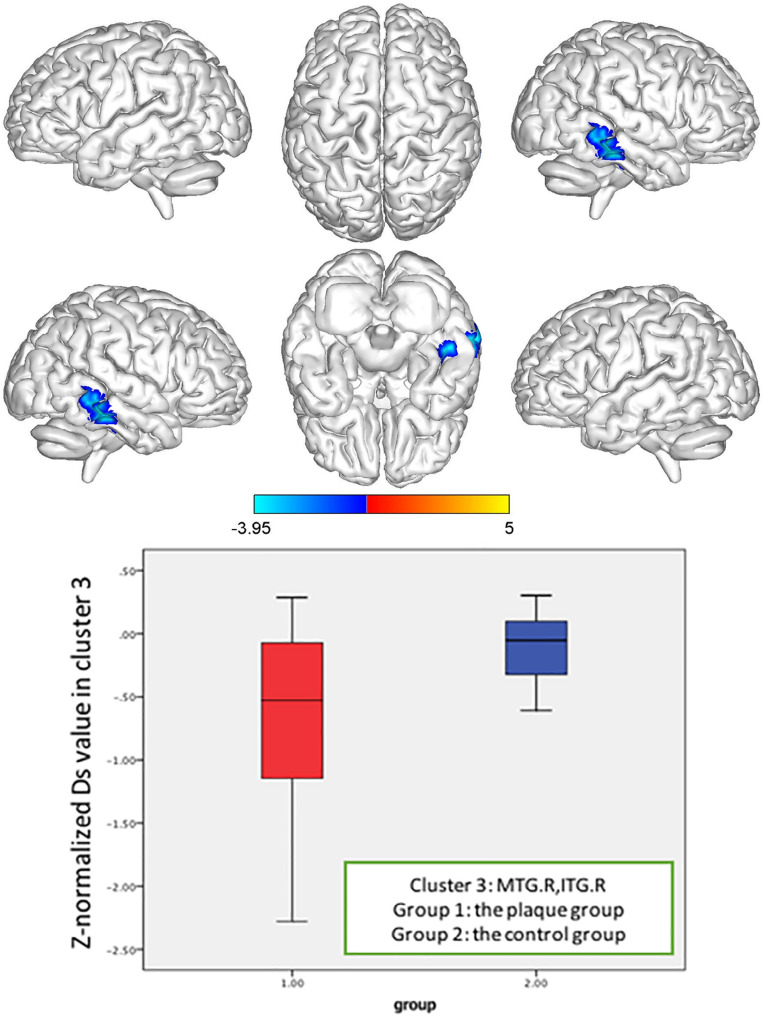
*Z*-normalized Ds value alterations in the asymptomatic carotid vulnerable plaque group. Significantly decreased Ds values in the right middle temporal gyrus (MTG.R) and inferior temporal gyrus (ITG.R) (cluster size = 116) (*p* < 0.05, FWE correction) compared to the control group.

**FIGURE 6:**
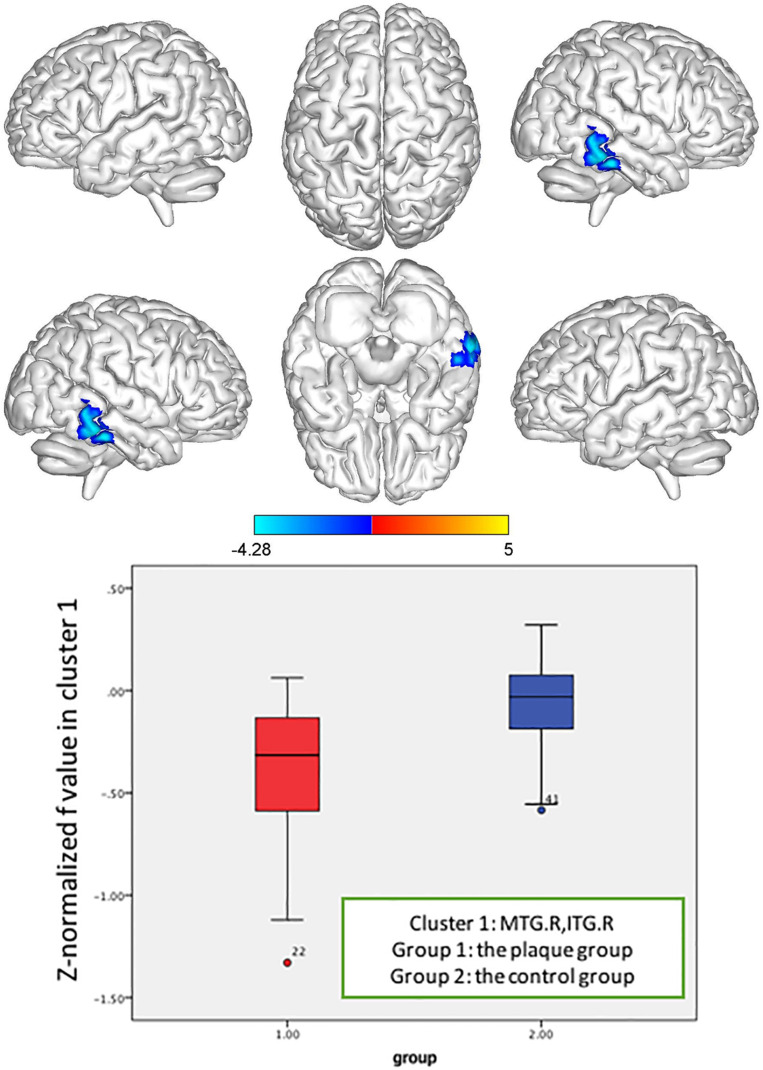
*Z*-normalized *f* value alterations in the asymptomatic carotid vulnerable plaque group. Significantly decreased *f* values in the right middle temporal gyrus (MTG.R) and inferior temporal gyrus (ITG.R) (cluster size = 85) (*p* < 0.05, FWE correction) compared to the control group.

Based on whole-brain voxelwise comparison, for patients with carotid vulnerable plaques but <50% stenosis, the *Z*-normalized Ds values were significantly higher than those of the control subjects in the left median cingulate and paracingulate gyrus (DCG.L), posterior cingulate gyrus (PCG.L) and left precuneus gyrus (PCUN.L) (cluster size = 156), and in the left middle frontal gyrus (MFG.L), orbital middle frontal gyrus (ORBmid.L), and superior frontal gyrus (SFG.L) (cluster size = 165). The *Z*-normalized Ds values were significantly lower in the right middle temporal gyrus (MTG.R) and inferior temporal gyrus (ITG.R) (cluster size = 116), and the *Z*-normalized *f*-values were significantly lower in the MTG.R and ITG.R (cluster size = 85) (*p* < 0.05, FWE correction) (Figures 4–6).

**FIGURE 7:**
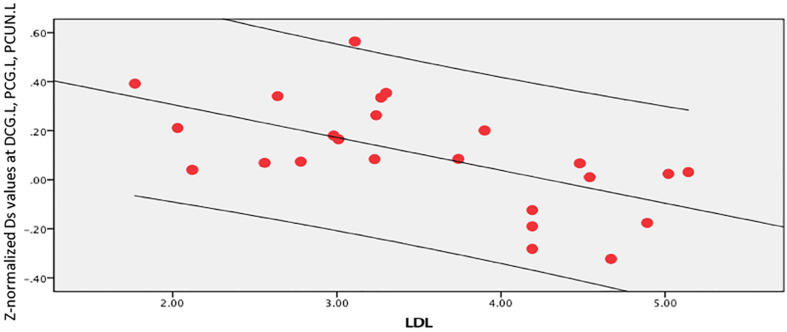
LDL-C was negatively correlated with Ds values in the left median cingulate and paracingulate (DCG.L), posterior cingulate gyrus (PCG.L), and left precuneus gyrus (PCUN.L) (*r* = 0.601, *p* = 0.002).

**FIGURE 8:**
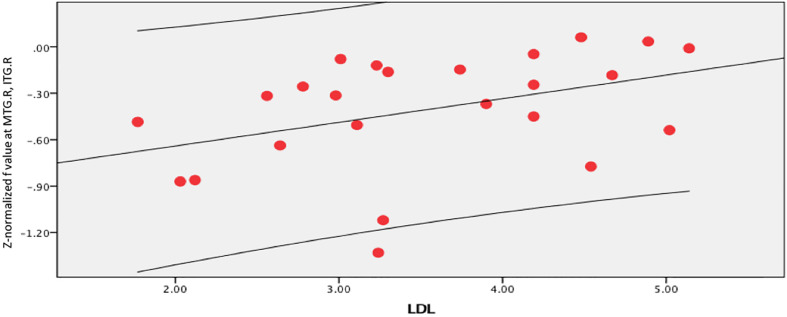
LDL-C was positively correlated with *f* value in the right middle temporal gyrus (MTG.R) and inferior temporal gyrus (ITG.R) (*r* = 0.405, *p* = 0.05).

**FIGURE 9:**
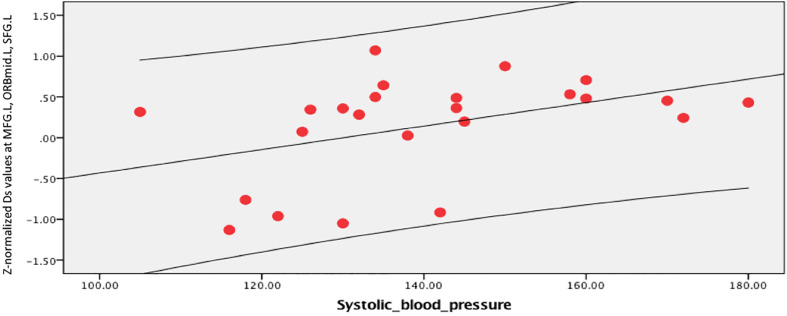
Systolic blood pressure was positively correlated with Ds values in the left middle frontal gyrus (MFG.L), orbital middle frontal gyrus (ORBmid.L), and superior frontal gyrus (SFG.L) (*r* = 0.433, *p* = 0.035).

In the brain regions with altered *Z*-normalized Ds values or *Z*-normalized *f*-values, LDL-C was negatively correlated with the Ds values in the DCG.DCGL, PCG.L, and PCUN.L (*r* = 0.601, *p* = 0.002). LDL-C was positively correlated with the *f*-values in the MTG.R and ITG.R (*r* = 0.405, *p* = 0.05). Systolic blood pressure was positively correlated with the Ds values in the MFG.L, ORBmid.L, and SFG.L (*r* = 0.433, *p* = 0.035) (Figures 7–9).

## Discussion

To the best of our knowledge, this is the first study to use IVIM combined with voxelwise comparisons based on a whole-brain spatial registration method to observe microcapillary perfusion and the brain microstructure in patients with carotid vulnerable plaques. We found that the cerebral regions with altered Ds values and *f* values in the carotid vulnerable plaque group were mostly related to cognition and emotion (e.g., the superior frontal gyrus, middle frontal gyrus, median cingulate and paracingulate gyrus, and posterior cingulate gyrus) and anoxia-sensitive regions (e.g., precuneus, middle temporal gyrus, and inferior temporal gyrus). Changes in microcapillary perfusion and the brain structure in patients with carotid vulnerable plaques showed no lateralization. Analysis of the blood and brain regions with altered Ds and *f* values showed that these parameters were partially related to LDL-C and systolic blood pressure, indicating the potential relationship between microvascular and microstructure alterations of the brain and vascular risk factors.

Previous studies have reported brain lateralization in whole-brain voxel-based morphometry (Gainotti, 2015). In the present study, first, the possible effect of the side with vulnerable plaques of carotid arteries was initially excluded because the stenosis of the carotid artery with unilateral plaques was less than 50%, which does not cause hemodynamic changes, and the circle of Willis could maintain cerebral hemodynamic stability (Liebeskind, 2003). Additionally, alterations in the *Z*-normalized Ds and *f* maps suggested that there was no lateralization in the changes in microcapillary perfusion and brain structure in patients with carotid vulnerable plaques.

Our study showed that the *Z*-normalized Ds values of the asymptomatic vulnerable carotid plaque group were significantly higher in the DCG.L, PCG.L, PCUN.L, MFG.L, ORBmid.L, and SFG.L at the cluster level. Ds is a diffusion coefficient, which represents the simple diffusion of water molecules. Possible reasons for an increase in Ds include decreased intracellular water content, increased cell membrane permeability, damage to the structural barrier, increased extracellular water ratio, widening of the extracellular space, and decreased limitation of water molecular activity, which represents damage to the microstructure of tissue.

Both the *Z*-normalized Ds and *Z*-normalized *f* values in the MTG.R and ITG.R of the asymptomatic vulnerable carotid plaque group were significantly lower than those of the control group at the cluster level. The decreased Ds values suggested that injury resulted in a decrease in extracellular water content, a decrease in cell membrane permeability, damage to the structural barrier, a decrease in the extracellular water ratio, a reduction in the extracellular space, and an increase in the water molecular activity, which reflected damage to tissue microstructures. The value of *f* represents the perfusion fraction, which indicates the proportion of related diffusion of microcirculatory perfusion in total diffusion of voxels and is related to blood volume. The decrease in the *f*-values suggested disorder of microperfusion in this region.

Alterations in the Ds values and *f*-values in the carotid vulnerable plaque group were mostly located in the regions related to cognition and emotion (e.g., SFG, MFG, DCG, and PCG) and anoxia-sensitive regions (e.g., PCUN, MTG, and ITG). Patients with carotid atherosclerosis with or without symptoms or increased carotid intima-media thickness often experience cognitive impairment and emotional symptoms (ESs) (Everts et al., 2014; Belem da Silva et al., 2019; Zhang et al., 2020). The SFG is thought to be involved in cognitive functions and emotion regulation-related processes, such as working memory and depression (Niendam et al., 2012), and low-frequency oscillation stimulation of the left SFG enhances working memory (Frank et al., 2014; Alagapan et al., 2019). It has been reported that perceived stress was positively correlated with fALFF in the left SFG and MFG, which play a partial mediating role in the relationship between perceived stress and depression (Wang et al., 2019). The DCG, PCG, and the precuneus are critical parts of the default mode network (DMN), which is related to cognition and emotion (Cavanna and Trimble, 2006; Leech and Sharp, 2014; Caruana et al., 2018). The ITG and MTG are associated with word comprehension-a cognitive process-in chronic post-stroke aphasia patients (Bonilha et al., 2017). A resting-state fMRI study showed synchronal abnormalities in the ITG and MTG of depressive patients who also have anxiety (Li et al., 2018). The ITG and MTG are also anoxia-sensitive regions, and hypoperfusion in the ITG and MTG was observed in amnestic mild cognitive impairment (aMCI), indicating impaired blood oxygen (Wang et al., 2020).

The Ds and f values in the altered brain areas were related to the LDL-C levels and systolic blood pressure. Smit et al. found that changes in the LDL-C levels were associated with lower cognitive impairment, reduced cerebral blood flow, and higher white matter hyperintensity in older people at different visits (Smit et al., 2016). LDL-C level and hypertension were associated with regional gray matter volume (GMV), and there was an interactive effect between low circulatory LDL-C level and hypertension (Chung et al., 2018). The explanation might be damage to the neurovascular unit (NVU), which causes neuronal degeneration and cognitive impairment (Zlokovic, 2011).

In the current study, alterations in the Ds and *f*-values were mainly distributed in the core brain areas of the combined cortex (e.g., the anterior cuneiform lobe, the superior frontal gyrus, and the middle frontal gyrus). This finding suggests that although there are no clinical symptoms in patients with asymptomatic carotid vulnerable plaques, the brain microstructure and microvessels changed at an early stage, and changes in the brain are complex and diffusive. Carotid atherosclerosis in asymptomatic populations was associated with brain structural changes and resting cerebral infarction (Romero et al., 2009; Cardenas et al., 2012). Increased carotid artery IMT and carotid stenosis ≥25% are related to a decrease in global brain volume, as well as white matter hyperintensity (Romero et al., 2009). These previous studies, together with this study, indicated that there was a relationship between asymptomatic carotid vulnerable plaques and subclinical brain injury, although the mechanism of this process is still unclear but may be explained by the following aspects. First, atherosclerosis is a systematic and complex pathological change that can occur simultaneously in multiple vessel beds, such as the carotid artery and cerebral artery (including microvessels) (Task Force Members et al., 2013; Wong et al., 2017). Second, vulnerable carotid plaques are a source of brain microemboli (Halliday et al., 2010) and are among the most important causes of sudden onset cerebral infarction (Ueda et al., 2010). Finally, inflammation might play a mediating role (Drakopoulou et al., 2011). Inflammatory cells are thought to play important roles in the occurrence and development of atherosclerosis, which is a type of inflammatory disease caused by multiple factors. Inflammation is the physiological basis of cognitive impairment. IVIM parameters can reflect subclinical brain alterations in patients with asymptomatic carotid vulnerable plaques and provide an imaging basis for the early diagnosis of asymptomatic carotid vulnerable plaques. Whether these altered diffusion and perfusion indices can be used as biomarkers for early warning of fatal or disabling diseases (e.g., stroke or cerebral vascular dementia) and their sensitivity and specificity will be the focus of our next study.

This study does have limitations. First, our sample size was small, which may obscure some subtle neuroimaging findings. Nevertheless, our study may allow hypothesis generation for future large-scale multicenter studies to identify the specific changes in the brains of people with asymptomatic carotid vulnerable plaques. Second, this was a single-center study, which may limit its generalization to asymptomatic carotid vulnerable plaque patients outside our institution. Third, we were limited by the cross-sectional design of this study, which could not determine the causes underlying the findings. We were also limited by lack of longitudinal follow-up data for these patients; however, as we said above, this will be included in our subsequent work.

## Conclusion

We suggest that IVIM should reflect subclinical brain alterations at an early stage in patients with asymptomatic carotid vulnerable plaques to a certain extent. Cerebral regions with altered IVIM parameters in the carotid vulnerable plaque group were mostly related to cognition and emotion, which could provide further insights into the potential association between carotid vulnerable plaques and cognition or emotion. IVIM might be utilized as a noninvasive biomarker of microvascular and microstructural changes in the brains of patients with asymptomatic carotid vulnerable plaques.

## Data Availability Statement

The raw data supporting the conclusions of this article will be made available by the authors, without undue reservation.

## Ethics Statement

The studies involving human participants were reviewed and approved by the ethical standards committee on human experimentation at Central South University (CSU). The patients/participants provided their written informed consent to participate in this study.

## Author Contributions

SY conceived the study and was in charge of overall direction and planning. LO analyzed the data. JG drafted the manuscript in consultation with SY. XW, WL, QH, WH, and GZ discussed the results and commented on the manuscript. All authors contributed to the article and approved the submitted version.

## Conflict of Interest

The authors declare that the research was conducted in the absence of any commercial or financial relationships that could be construed as a potential conflict of interest.

## Footnotes

1 http://www.fil.ion.ucl.ac.uk/spm/software/spm12/
